# Therapeutic Lifestyle Strategies Taught in U.S. Pharmacy Schools

**Published:** 2007-09-15

**Authors:** Thomas L Lenz, Michael S Monaghan, Elizabeth A Hetterman

**Affiliations:** Department of Pharmacy Practice, Creighton University Medical Center; Creighton University Medical Center, Omaha, Nebraska; Creighton University Medical Center, Omaha, Nebraska

## Abstract

**Introduction:**

Several organizations representing pharmacy and other health professions stress the importance of teaching public health topics as part of training future practitioners. The objective of our study was to assess the number of U.S. pharmacy schools that incorporate lifestyle modification topics into their curricula.

**Methods:**

We developed an electronic survey on lifestyle modification topics and sent it to each of the 89 pharmacy schools in the United States. The survey defined lifestyle modification topics as topics that address nutrition, exercise, weight loss, smoking cessation, and alcohol use.

**Results:**

Of 89 pharmacy schools contacted, 50 (56%) responded to the survey. Of the 50, four offer at least one required course in a lifestyle modification topic, seven offer at least one elective course, and one offers a required course that incorporates more than one lifestyle modification topic. Five required and nine elective courses were identified from the responses. Nutrition was the most commonly offered required course topic, followed by smoking cessation, exercise, weight loss, and alcohol use.

**Conclusion:**

Few pharmacy schools are addressing recommendations to promote public health education through formalized didactic courses. More courses on lifestyle modification topics should be offered to pharmacy students, who will be highly accessible to the public as pharmacists and will be able to offer education to enhance public health focused on the prevention of chronic diseases.

## Introduction

Health promotion, wellness, and disease prevention are becoming increasingly important for health care professionals. A significant amount of data exist to demonstrate that population health can be improved in a cost-effective manner by preventing diseases rather than treating them after they occur (1-3). Unfortunately, traditional training for health care professionals does not focus on disease prevention, but rather on treating diseases once they occur. In an effort to change the landscape of the formal training of student health care professionals, several organizations now recommend curriculum changes to include courses about health promotion, wellness, and disease prevention (3).

In 2002 the Healthy People Curriculum Task Force was established with the mission of fulfilling objective 1-7 of *Healthy People 2010* (2,4), to "increase the proportion of schools of medicine, schools of nursing, and other health professional training schools whose basic curriculum includes the core competencies in health promotion and disease prevention" (2). In 2004, the task force published a curriculum framework for health professions in clinical prevention and population health (3). Seven health professional organizations participated in the project, including one representing pharmacy.

In addition to the Healthy People Curriculum Task Force, pharmacy organizations such as the American Association of Colleges of Pharmacy (AACP) have made curriculum recommendations to pharmacy schools through the Center for the Advancement of Pharmaceutical Education (CAPE) *Educational Outcomes 2004* (5). One of the three major CAPE educational outcomes recommendations is the teaching of public health with an emphasis on promoting health improvement, wellness, and disease prevention (5). The American Society of Health-Systems Pharmacists has created the 2015 Initiative (http://www.ashp.org/2015) with six primary goals. Goal number 6 is to "increase the extent to which pharmacy departments in health systems engage in public health initiatives on behalf of their communities." Additionally, the American Pharmacists Association has sponsored studies to show that lifestyle modification strategies can be incorporated into a pharmacy practice setting and be both clinically effective and cost-effective. (6,7).

It is unknown how many schools of pharmacy in the United States currently offer their students courses on lifestyle modification specifically focused on health promotion, wellness, and disease prevention. The primary objective of our study was to determine this.

## Methods

We developed an electronic survey specifically for this study to gather the necessary information to answer our research questions. We employed a table of specifications for survey development. The survey defined health promotion, wellness, and disease prevention as programs that offer courses specifically focused on nutrition for healthy living, exercise programs, weight loss, smoking cessation, appropriate alcohol use, or a combination of these topics. The survey consisted of six questions, two of which included a table for the responders to complete. The survey was designed and administered on Microsoft FrontPage 2003 software (Microsoft Corporation, Redmond, Washington).

Survey work began in February 2005 with identification of the chair of the curriculum committee at each of the 89 U.S. pharmacy schools in existence at that time via a telephone conversation with a school administrator. Information obtained included each chair's name, mailing address, telephone number, and e-mail address. We mailed a short letter to each committee chair in April 2005 to explain the study and to inform the chairs that the survey would be sent to them via e-mail in approximately 1 week. Ten days later we sent the electronic survey via e-mail to each chair, and we followed this initial mailing with two reminder e-mails to those who did not respond. The Creighton University Institutional Review Board reviewed this study before data collection and considered it exempt from the Federal Policy for Protection of Human Subjects.

## Results

Fifty of the 89 survey recipients responded, yielding a 56% response rate. Of these respondents, four schools offered at least one required course. Three schools required a course on nutrition, and one school required a course on smoking cessation. One respondent reported offering a required course to students that incorporated more than one lifestyle topic in the same course. Another seven respondents reported that their schools offered at least one elective course solely devoted to one of the five topics: three schools offered a course on exercise; two schools, nutrition; two schools, smoking cessation; one school, weight loss; and one school, alcohol use.

## Discussion

Chronic diseases impose a significant societal, personal, and financial burden on the U.S. health care system (8). The Centers for Disease Control and Prevention (CDC) reports that 70% of U.S. deaths and 75% of the nation's annual health care costs are related to chronic diseases (8). These data indicate that a focus on the prevention of these chronic diseases may produce better overall outcomes for patients and be cost-effective for the U.S. health care system.

Consumers have long recognized pharmacists as highly accessible and well-trusted health care providers (9). In addition, many of the most commonly dispensed drugs in pharmacies are used to treat or prevent conditions in which lifestyle changes such as physical activity, proper nutrition, weight loss and control, and smoking abstinence are also recommended to prevent the disease (10). Because of these factors, pharmacists are in an ideal position to offer patients information, guidance, and counseling on lifestyle changes that can help them manage their medical conditions.

In one study, researchers showed that although patients with dyslipidemia visit the pharmacy more often than the physician's office, pharmacists offer less information to patients newly diagnosed with dyslipidemia about lifestyle changes than do physicians and nurses (11). This same study also reported that patients with dyslipidemia receive more follow-up lifestyle modification information to help control dyslipidemia from their physician or nurse, a dietitian, and even the media than from their pharmacist (11). One reason for the lack of this type of counseling by pharmacists may be inadequate knowledge, skills, and confidence to properly counsel patients on lifestyle changes. This knowledge and these skills can be acquired through course work before graduation.

As previously stated, the Healthy People Curriculum Task Force and several other organizations recommend that schools that train student health care professionals include core competencies in health promotion and disease prevention in their required curricula. The gap between the pharmacy curricula and this recommendation most likely exists for several reasons, including 1) a traditional patient care strategy that focuses on treating rather then preventing illness; 2) a lack of individuals qualified to teach topics related to health promotion, wellness, and disease prevention; 3) an attitude among pharmacy professionals that preventive care is the responsibility of another health care provider and is therefore not needed within the pharmacy curriculum; 4) a lack of space within the current curricula for preventive care courses; and 5) the concern that practicing pharmacists do not have time to talk with patients about disease prevention and, furthermore, are not reimbursed for doing so. A refocus of the patient care model to one of prevention, along with pharmacist reimbursement for disease prevention counseling, could significantly help U.S. pharmacy schools adopt curricula that require courses focused on lifestyle modification strategies. Also important is emphasis on the pharmacist's role as a member of the patient's health care team who communicates both with the patient and with other health care professionals.

Our study showed that few U.S. pharmacy schools offer student pharmacists the opportunity to gain knowledge and skills about lifestyle changes that promote health and wellness and prevent disease. Several organizations representing pharmacy and nonpharmacy health professions recommend that all health care professionals in training, including student pharmacists, receive formal education on health promotion and disease prevention. Pharmacy education has more frequently adopted immunization training for students in recent years as a way to prevent disease and improve public health than it has lifestyle modification. Pharmacy schools have been slow to incorporate other disease prevention strategies into their curricula, however, as is evident from our study. More U.S. pharmacy programs should work to adopt curricula recommended by AACP that offer more emphasis on public health and include health promotion, wellness, and disease prevention.

Although we accomplished the original objective established for our study, there are a few limitations. We wanted to achieve at least a 60% response rate (53 of 89 potential respondents) and fell short by three respondents. The time of year coincided with semester end and final exams and may have had an impact on the response rate. Nonetheless, we feel confident that the data collected are representative of the current state of pharmacy education, and we believe it is unlikely that a significant number of the nonresponders have heavy focus on lifestyle modification courses in their curricula. An additional limitation is that lifestyle modification topics may be discussed as a part of other courses in the pharmacy curriculum. These topics may not receive major emphasis for that particular course, but students may be gaining some knowledge through this avenue. We attempted to gather this type of information in our study but were unable to do so. Another limitation is that some students may have gained information in this area by taking elective courses outside the pharmacy program curriculum.

## Conclusion

Pharmacists and other health care professionals need the knowledge and skills required to promote health, wellness, and disease prevention to adequately manage chronic diseases. Several pharmacy and nonpharmacy organizations recommend that pharmacy curricula include courses that give students this knowledge and these skills. Our study found that only a small percentage of pharmacy schools offer elective courses to address this need, and even fewer offer required courses on therapeutic lifestyle strategies. The AACP recommendations for pharmacy schools state that health promotion and disease prevention should be a major part of a student pharmacist's didactic training. More U.S. pharmacy programs should offer their student pharmacists the opportunity to gain knowledge and skills to promote health, wellness, and disease prevention so these future pharmacists can adequately work with patients with chronic disease. Not only will such education enhance the student's clinical skills, but it will also enhance the pharmacist's role as a health care professional devoted to total health care and not simply to dispensing medications.

## References

[1] Mokdad AH, Marks JS, Stroup DF, Gerberding JL (2004). Actual causes of death in the United States, 2000. JAMA.

[2] U.S. Deaprtment of Health and Human Services. Healthy People 2010.

[3] Allan J, Barwick TA, Cashman S, Cawley JF, Day C, Douglass CW (2004). Clinical prevention and population health: curriculum framework for health professions. Am J Prev Med.

[4] Allan J, Barwick TA, Cawley JF, Day C, Douglass CW, Evans CH (2004). Clinical prevention and population health: curriculum framework for health professions. Am J Prev Med.

[5] Nemire RE, Meyer SM (2006). Educating students for practice: educational outcomes and community experience. Am J Pharm Educ.

[6] Bluml BM, McKenney JM, Cziraky MJ (2000). Pharmaceutical care services and results in project ImPACT: hyperlipidemia. J Am Pharm Assoc (Wash).

[7] Bunting BA, Cranor CW (2006). The Asheville Project: long-term clinical, humanistic, and economic outcomes of a community-based medication therapy management program for asthma. J Am Pharm Assoc (2003).

[8] Hardy GE (2004). The burden of chronic disease: the future is prevention. Prev Chronic Dis.

[9] Knapp KK (1999). Charting the demand for pharmacists in the managed care era. J Am Pharm Assoc (Wash).

[10] RxList Inc. Top 300 drugs of 2005 by number of US prescriptions dispensed.

[11] Lenz TL, Stading JA (2005). Lifestyle modification counseling of patients with dyslipidemias by pharmacists and other health professionals. J Am Pharm Assoc (2003).

